# Antimicrobial and Efflux Pump Inhibitory Activity of Caffeoylquinic Acids from *Artemisia absinthium* against Gram-Positive Pathogenic Bacteria

**DOI:** 10.1371/journal.pone.0018127

**Published:** 2011-04-04

**Authors:** Yiannis C. Fiamegos, Panagiotis L. Kastritis, Vassiliki Exarchou, Haley Han, Alexandre M. J. J. Bonvin, Jacques Vervoort, Kim Lewis, Michael R. Hamblin, George P. Tegos

**Affiliations:** 1 Laboratory of Analytical Chemistry, University of Ioannina, Ioannina, Greece; 2 Bijvoet Center for Biomolecular Research, Science Faculty, Utrecht University, Utrecht, The Netherlands; 3 NMR Center, University of Ioannina, Ioannina, Greece; 4 Wellman Center for Photomedicine, Massachusetts General Hospital, Boston, Massachusetts, United States of America; 5 Wageningen NMR Center, Laboratory of Biophysics, Wageningen, The Netherlands; 6 Department of Biology and Antimicrobial Drug Discovery Center, Northeastern University, Boston, Massachusetts, United States of America; 7 Department of Dermatology, Harvard Medical School, Boston, Massachusetts, United States of America; 8 Harvard-MIT Division of Health Sciences and Technology, Cambridge, Massachusetts, United States of America; Deutsches Krebsforschungszentrum, Germany

## Abstract

**Background:**

Traditional antibiotics are increasingly suffering from the emergence of multidrug resistance amongst pathogenic bacteria leading to a range of novel approaches to control microbial infections being investigated as potential alternative treatments. One plausible antimicrobial alternative could be the combination of conventional antimicrobial agents/antibiotics with small molecules which block multidrug efflux systems known as efflux pump inhibitors. Bioassay-driven purification and structural determination of compounds from plant sources have yielded a number of pump inhibitors which acted against gram positive bacteria.

**Methodology/Principal Findings:**

In this study we report the identification and characterization of 4′,5′-*O*-dicaffeoylquinic acid (4′,5′-*O*DCQA) from *Artemisia absinthium* as a pump inhibitor with a potential of targeting efflux systems in a wide panel of Gram-positive human pathogenic bacteria. Separation and identification of phenolic compounds (chlorogenic acid, 3′,5′-*O*DCQA, 4′,5′-*O*DCQA) was based on hyphenated chromatographic techniques such as liquid chromatography with post column solid-phase extraction coupled with nuclear magnetic resonance spectroscopy and mass spectroscopy. Microbial susceptibility testing and potentiation of well know pump substrates revealed at least two active compounds; chlorogenic acid with weak antimicrobial activity and 4′,5′-*O*DCQA with pump inhibitory activity whereas 3′,5′-*O*DCQA was ineffective. These intitial findings were further validated with checkerboard, berberine accumulation efflux assays using efflux-related phenotypes and clinical isolates as well as molecular modeling methodology.

**Conclusions/Significance:**

These techniques facilitated the direct analysis of the active components from plant extracts, as well as dramatically reduced the time needed to analyze the compounds, without the need for prior isolation. The calculated energetics of the docking poses supported the biological information for the inhibitory capabilities of 4′,5′-*O*DCQA and furthermore contributed evidence that CQAs show a preferential binding to Major Facilitator Super family efflux systems, a key multidrug resistance determinant in gram-positive bacteria.

## Introduction

Multi-drug resistant microbial infections caused by Gram-positive bacteria such as *Staphylococcus aureus* and *Enterococcus faecalis* represent an exponentially growing problem affecting communities worldwide. Efflux mechanisms have become broadly recognized as major components of resistance to many classes of antibiotics [1], [2]. Some efflux pumps selectively extrude specific antibiotics while others, referred to as multidrug efflux pumps (MEPs), expel a variety of structurally and functionally diverse compounds [3] in addition to a variety of other physiological roles [4]. A novel and promising approach to deal with multidrug resistance is to improve the clinical performance of various antibiotics by employing efflux pump inhibitors (EPIs) [5], [6]. Plants have been explored comprehensively as potential sources of antimicrobials [7], [8]. It has been established that disabling MEPs in Gram-negative species using a combination of MEP mutants and synthetic EPIs leads to a striking increase in the activity of numerous plant compounds [9]. Several *Berberis* medicinal plants (*Berberis repens*, *B. aquifolia*, and *B. fremontii*) that produce the plant antimicrobial berberine also synthesized an inhibitor of the *Staphylococcus aureus* NorA MEP identified as 5′-methoxyhydnocarpin (5′-MHC) [10]. Identification of 5′-MHC intensified the search for natural EPIs of plant origin. Bioassay driven purification and structural determination of compounds from various plant sources yielded a number of EPIs acting against Gram- positive bacteria with activities similar to 5′-MHC [11], [12], [13], [14], [15], [16]. Gram-negative bacteria and fungi comprise the main groups of plant pathogens, and it is important to determine whether the antimicrobial/EPI synergy is a general mechanism of plant defense.

*Artemisia* is a fairly large genus within the family of the *Asteraceae* (*Compositae*), with more than 200 individual species [17]. *Artemisia absinthium,* best known as the principal ingredient in the infamous Absinthe drink, has been used medicinally since the times of ancient Greece, and also in western European systems of traditional medicine [17]. It was recently reported that the essential oils occurring in flowers and aerial parts from *A. absinthium* have antimicrobial properties [17]. Moreover aqueous extracts of *A. absinthium* are rich in caffeoyl and dicaffeoylquinic acids, which are known to inhibit HIV-1 integrase from integrating the reversibly transcribed viral DNA into host cell DNA [18]. Furthermore, these components are hepatoprotective, anti-histaminic, hypocholesterolemic, anti-spasmodic, and potentially antimicrobial. They also demonstrate high antioxidant activity [19] due to their o-quinone moieties. Caffeoylquinic acids could inhibit oxidative damage to both low-density lipoprotein [20] and linolenic acid [21] by scavenging reactive oxygen species (ROS) related to cancer and cardiovascular diseases. It has been reported that chlorogenic acid (5-*O*-caffeoylquinic acid) had inhibitory effects on carcinogenesis in the large intestine, liver, and tongue, and exhibited protective effects on oxidative stress *in vivo* [22].

The effort to discover plant EPIs, a process ranging from identifying a hit to isolating a pure compound, presents a distinct bottleneck: the lack of comprehensive, chemically and biologically representative natural product extract libraries. Analysis of natural product extracts is time-consuming and laborious, consisting of the isolation of different components of the extract and the successive identification of these compounds by spectrometric data acquisition. We have identified EPI activity against Gram-positive bacteria in *A. absinthium* extracts. We dramatically expedited the analysis of *A. absinthium* aquatic infusions using analytical techniques (LC-SPE-NMR and LC-MS), revealing caffeoylquinic acids as key components. Subsequent antimicrobial susceptibility testing of berberine uptake and a competitive efflux assay employing the pure compounds was conducted. An array of pathogens, including knock out and over expression MEP mutants, strongly supported the hypothesis that 4′,5′-*O*DCQA behaves as a designated EPI for the major facilitator super family (MFS) MEPs of the Gram-positive bacteria: *S. aureus*, and *Enterococcus faecalis*.

## Results and Discussion

### Antimicrobial and EPI activity of extracts

The experimental approach to detect EPI activity was to test the combined action of a plant extract (the no alkaloid fraction) with berberine added at a sub inhibitory concentration. Extracts that inhibited cell growth in the presence of berberine and had no activity when added alone were likely to contain an EPI. Chloroform extracts of leaves from *A. absinthum* had no antimicrobial activity alone at up to 500 µg/ml (**Table 1**), but inhibited *Staphylococcus aureus*, *Enterococcus faecalis* and *Bacillus cereus* growth completely in the presence of 30 µg/ml berberine (15 µg/ml berberine for *Bacillus* cells), a concentration one-eighth the MIC for this substance (data not shown).

**Table 1:** Antimicrobial susceptibility for *A. absinthum* and acquired CQA derivatives.

	MIC (µg ml^−1^)
Strain	Extr	3′,5′-*O*DCQA	4′,5′-*O*DCQA	5′-*O*CQA	1′,3′-*O*DCQA	1′,5′-*O*DCQA
S. aureus 8325-4	256	>128	>128	128	>128	>128
*norA*	256	>128	>128	64	>128	>128
*NorA++*	256	>128	>128	>128	>128	>128
CA-MRSA	256	>128	>128	>128	>128	>128
*B. cereus*	128	>128	>128	64	>128	>128
*E. faecalis*	256	>128	>128	64	>128	>128
*norA*	256	256	>256	32	>256	>256
*E. coli*	>256	>256	>256	>256	>256	>256
*C. albicans*	>256	>256	>256	>256	>256	>256

All MIC determinations were performed in triplicate.

The three main components of the extract consisting 40% of the total mass were identified via LC-UV-SPE-NMR [23] and LC-MS. The molecular ion in the ESI negative mode of the first peak was m/z  = 353 and the MS^2^ gave a characteristic chlorogenic acid fragmentation pattern [24]. The structure was confirmed by the respective ^1^H-NMR analysis. The second and third main components of the extract were co-eluted from the chromatographic column. By the spectral data obtained it was concluded that two dicaffeoylquinic acid isomers were present (3′,5′-*O*DCQA and 4′,5′-*O*DCQA) (**Figures S1, S2**).

### Antimicrobial and EPI activity of CQAs

The *A. absinthum* extract was tested against a panel of human and plant pathogenic bacteria both for direct antimicrobial and EPI activity. The extract exhibited neither antimicrobial activity nor EPI activity in *Escherichia coli* or *Candida albicans* (concentration range 150–500 µg/mL). It showed no apparent direct antimicrobial activity (MICs in the range of 128–256 µg/mL for all Gram-positive bacteria tested), but completely inhibited growth in concentrations lower than 50 µg/mL when berberine was added in sub inhibitory concentrations (30 µg/mL for *S. aureus and E. faecalis*, and 15 µg/mL for *B. cereus*).

We obtained 5′-*O*-caffeoylquinic (5′-*O*CQA) acid and peracetylderivatives 1′,3′- and 1′,5′-*O*DCQA, 3′,5′-*O*DCQA and 4′,5′-*O*DCQA, and tested their direct antimicrobial and EPI activity (**Table 1**). 5′-*O*CQA was the only molecule with weak direct antimicrobial activity against the Gram-positive bacteria (*B. cereus* and *E. faecalis*, MIC 64 µg/ml). There was a 4-fold difference between MICs in *S. aureus* 8325-4 and the isogenic NorA mutant providing supporting evidence for 5′-*O*CQA as a potential substrate of MFS efflux systems. All other derivatives were virtually inactive with significantly higher MICs (>128 µg/ml) against all microorganisms in the panel tested.

### Toxicity studies

**Figure 1:**
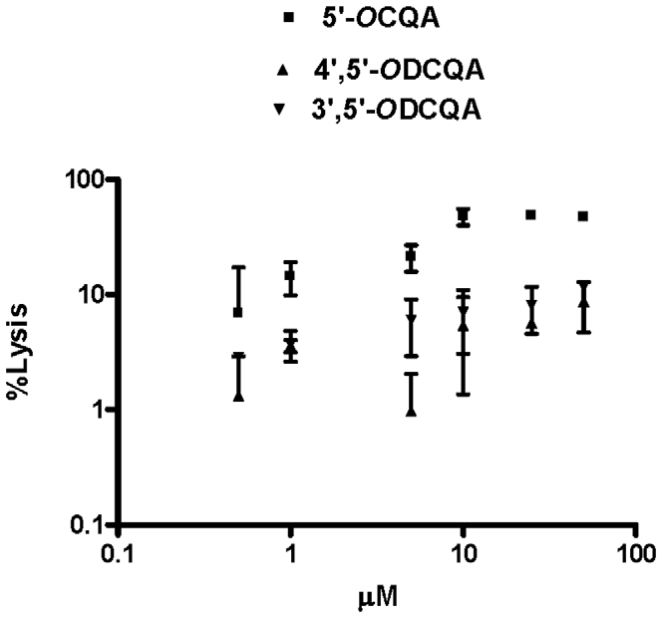
Hemolytic activity of caffeoylquinic acids towards sheep erythrocytes. The CQA induced significantly more (*) hemolysis, while DCQAs induced significantly virtually no hemolysis. The data were analyzed by two-way repeated-measurement ANOVA for the factors of different derivatives and derivative concentrations followed by post hoc testing by the Holm-Sidak method (*P*<0.05). Values are means ± SEM (*n* = 3). Note the log scales on both the *x* and *y* axes.

Hemolysis experiments using sheep erythrocytes and DCQA derivatives demonstrated no hemolytic activity at 50 µM, a concentration higher than the MICs for Gram-positive and significantly higher than the concentration used for antimicrobial potentiation (**Figure 1**). There are a few brief reports discussing the toxic effect of 5′-*O*CQA on human oral squamous cell carcinoma (HSC-2) and salivary gland tumor (HSG) cell lines, as compared with that against human gingival fibroblast (HGF), and erythrocytes in a mM range [25]. The toxic effect is attributed to the potential of the compound to generate reactive oxygen species (ROS). A dose-response experiment using erythrocytes was also able to detect toxicity of 5′-*O*CQA at fairly low concentrations, (1-5 µM) whereas 3′,5′- and 4′,5′-*O*DCQAs did not exhibit any cell-lytic properties at significantly higher concentrations (50 µM). This trend, together with existing studies [22], [26], suggest that 5′-*O*CQA is toxic at high concentrations, and implies that the other derivatives appear to have a different interaction pattern with mammalian cells, suggesting that a therapeutic window may exist in both cases. 3′,5′-*O*DCQA and 4′,5′-*O*DCQA showed extremely low toxicity to the blood cells indicating that the antimicrobial activity and toxicity patterns are relatively similar related to the differences in substitution moieties.

### Potentiation of antibiotics by 4′,5′-*O*DCQA

We conducted a checkerboard assay using berberine and CQAs against *S. aureus* 8325-4 (**Table 2**). This method supports evidence for synergy, competition or antagonism for selected compounds as it is reflected by the FIC index (FICI). A FICI of ≤0.5 indicates synergistic interaction. FICI is calculated as the sum of the FICs of each agent. The FIC of each agent is calculated as the MIC of the agent in combination divided by the MIC of the agent. This indicated a synergistic effect for 4′,5′-*O*DCQA, an′d competition for the other two derivatives. We employ a clinical MRSA strain (CA-300) and conducted extensive checkerboard assays using an array of known MFS substrates and 4′,5′-*O*DCQA suggesting a promising range of activity both in different strains and clinically relevant compounds (**Table 3** **)**. We further evaluated the ability of 4′,5′-*O*DCQA to potentiate designated substrates of MFS and known antibiotics against a panel of Gram-positive phenotypes (**Tables 4** **,** **5**). The activity of all of the MFS substrates increased significantly in the presence of the compound against the wild-type strains tested (8-fold for berberine, and 4-8-fold for EtBr and fluroquinolones). The addition of 4′,5′-*O*DCQA in the more susceptible *E. faecalis* NorA knock out mutant (**Table 5**) increased the susceptibility of EtBr and norfloxacin (2–4 fold). This result indicated the possible additional participation of the QacA MEP system which has been implicated in the efflux of NorA substrates [12], [27]. Besides this anticipated complication, the ability of 4′,5′-*O*DCQA to potentiate antibiotics was significantly weaker in the case of the NorA knock out phenotypes, indicating a likely mode of action associated with this MFS efflux system. Moreover the *S. aureus* with NorA overexpression showed significant potentiation of all antimicrobials and gave susceptibilities that were comparable with the wild type counterpart in the absence of 4′,5′-*O*DCQA (8-fold for berberine, between 4- 8-fold for EtBr and fluroquinolones). Results were similar when a *B. cereus* were used (data not shown), implying a wider range of activity for 4′,5′-*O*DCQA.

**Table 2:** Checkerboard assay for interaction of DCQAs with BERB against *S. aureus.*

Compound	FIC (compound)	FIC (BERB)	FICI
5′-*O*CQA	0.50	0.50	1.00
3′,5′-*O*DCQA	1.00	1.00	1.00
4′,5′-*O*DCQA	0.125	0.0625	0.5

**Table 3:** Checkerboard assay of 4′,5′-*O*DCQA with antimicrobials against MRSA.

Antibiotic (Dye)	FIC (antibiotic or dye)	FIC (4′,5′-*O*DCQA)	FICI
CIPRO	0.20	0.00078	0.0039
NOR	0.10	0.00039	0.0039
LEVO	0.40	0.00156	0.0039
BERB	0.0312	0.0625	0.50
EtBr	0.0625	0.0156	0.0625

a Evaluated by the checkerboard method recommended by the CLSI and expressed as the FIC index (FICI). A FICI of ≤0.5 indicates synergistic interaction. FICI is calculated as the sum of the FICs of each agent. The FIC of each agent is calculated as the MIC of the agent in combination divided by the MIC of the agent alone.

**CIPRO**, ciprofloxacin **NOR**, norfloxacin **LEVO**, levoflaxin **BERB**, bebrerine, **EtBr**, Ethidium Bromide.

**Table 4:** Potentiation of antimicrobials by 4′,5′-*O*DCQA in *S. aureus.*

	*S. aureus 8325-4*		NorA −/− *S. aureus* (MIC µg/mL)		NorA+/+ *S. aureus*	
	alone	+4′,5′-*O*DCQA	alone	+4′,5′-*O*DCQA	alone	+4′,5′-*O*DCQA
BERB	256	16	16	8	>512	64
EtBr	64	4	8	8	256	64
CIPRO	1	0.2	0.4	0.8	1	0.4
NOR	1	0.1	0.2	0.2	2	0.8
MOXI	1	0.2	0.4	0.4	1	0.4
LEVO	1	0.4	0.8	0.4	4	1

The strains *S. aureus* 8325-4 (wt) and the isogenic pair of knock out and overexpression NorA mutant were used. All MIC determinations were performed in triplicate. * **4′,5′-** ***O*** **DCQA** was added at a final concentration of 10 µg/mL.

**MOXI,** moxifloxacin.

**Table 5:** Potentiation of antimicrobials by 4′,5′-*O*DCQA in *E. faecalis*.

	OGRF1 (MIC µg/mL)		norA−/− *E. Faecalis* (MIC µg/mL)	
	alone	+4′,5′-*O*DCQA	alone	+4′,5′-*O*DCQA
BERB	512	64	32	16
EtBr	128	8	16	4
CIPRO	2	0.8	1	1
NOR	1	0.2	0.4	0.2
MOXI	1	0.1	0.2	0.4
LEVO	1	0.1	0.2	0.1

The strains OGRF1(wt) and the knock out NorA mutant were used. All MIC determinations were performed in triplicate. **4′,5′-** ***O*** **DCQA** was added at a final concentration of 10 µg/mL.

### 4′,5′-*O*DCQA enhances accumulation of berberine in Gram-positive bacteria

**Figure 2:**
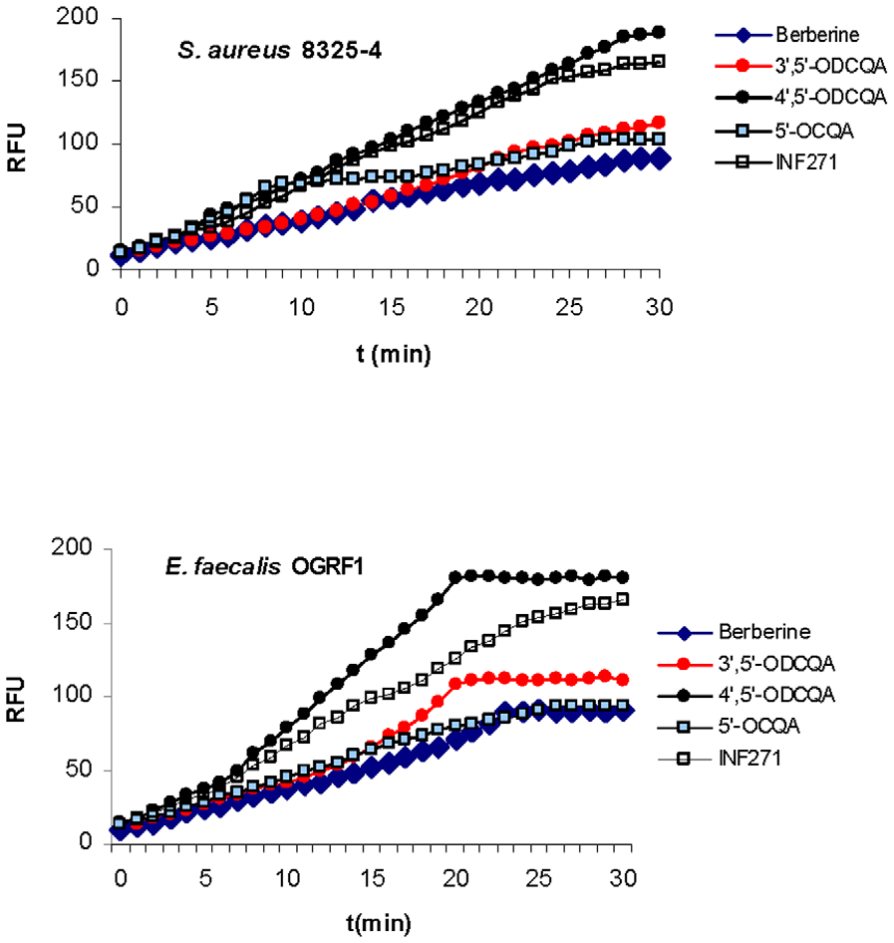
Berberine accumulation with caffeoylquinic acids in Gram-positive bacteria. The uptake of berberine with no addition or with the addition of 5′-*O*CQA, 3′,5′-*O*DCQA, 4′,5′-*O*DCQA, or INF_271_ by cells of *S. aureus* (A), *E. faecalis* (B), was measured by determination of the increase in fluorescence following binding to DNA and is expressed as relative fluorescence units (RFU). Berberine was present at a concentration of 30 µg/ml, and 5′-*O*CQA, 3′,5′-*O*DCQA, 4′,5′-*O*DCQA, INF_271_ were added at the same final concentrations used for the MIC determinations.

Berberine is a useful model plant antimicrobial whose accumulation in microbial cells can be conveniently monitored by measuring the fluorescence of berberine bound to DNA We used three different Gram-positive bacteria, *S. aureus*, *E. faecalis*, and *B. cereus*, to evaluate with a functional assay the EPI potential of CQAs. We monitored an increase in berberine fluorescence, assessing the rate of penetration and the level of accumulation of berberine in *S. aureus* cells, in response to an established NorA inhibitor (INF_271_) [28], [29] and the selection of CQA derivatives (**Figure 2A** **)** compared to berberine alone. Rapid accumulation was also observed in other strains tested (*E. faecalis,* **Figure 2B and ***B* *. cereus* data not shown). In all cases the potentiation of uptake was maximal for 4′,5′-*O*DCQA. The effect was equal or higher than that seen with INF_271_. Accumulation of berberine was observed in the presence of 3′,5′-*O*DCQA and 5′-*O*CQA, although the rate and level of uptake were lower compared with 4′,5′-*O*DCQA, and closer to the baseline of berberine. This data agrees with the EPI activity and the checkerboard assay data.

**Figure 3:**
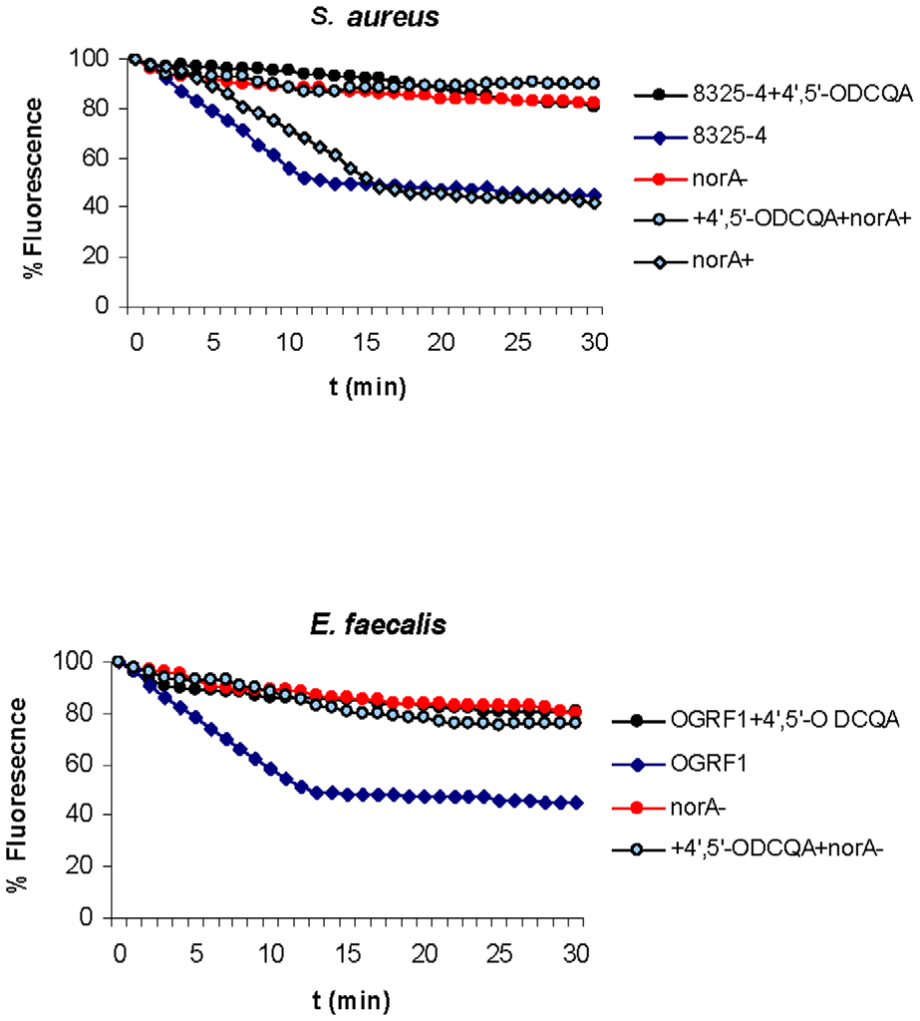
Efflux potentiation by 4′,5′-*O*-dicaffeoylquinic acid in *S. aureus* (A), *E. faecalis* (B). Efflux was measured using berberine by determination of the decrease in fluorescence following binding to DNA and is expressed as % fluorescence. Cells were loaded with berberine at a concentration of 100 µg/ml, and then resuspended in buffer in the absence or presence of 4′,5′-*O*DCQA, at the same final concentrations used for the MIC determinations.

To study efflux, we loaded cells with berberine or the combination of berberine with the compound at concentrations significantly higher than those used for antimicrobial susceptibility testing and then transferred them into fresh medium. **Figure 3A** (*S. aureus)* and **Figure 3B** (*E. faecalis)* show that berberine efflux was inhibited in the presence of 4′,5′-*O*DCQA both in wild type strains as well as the *S. aureus* NorA overexpressing strain. The lack of any effect in NorA knockout strains provides additional evidence that 4′,5′-*O*DCQA is inhibiting NorA.

### Biofilm inhibition with CQAs

**Figure 4:**
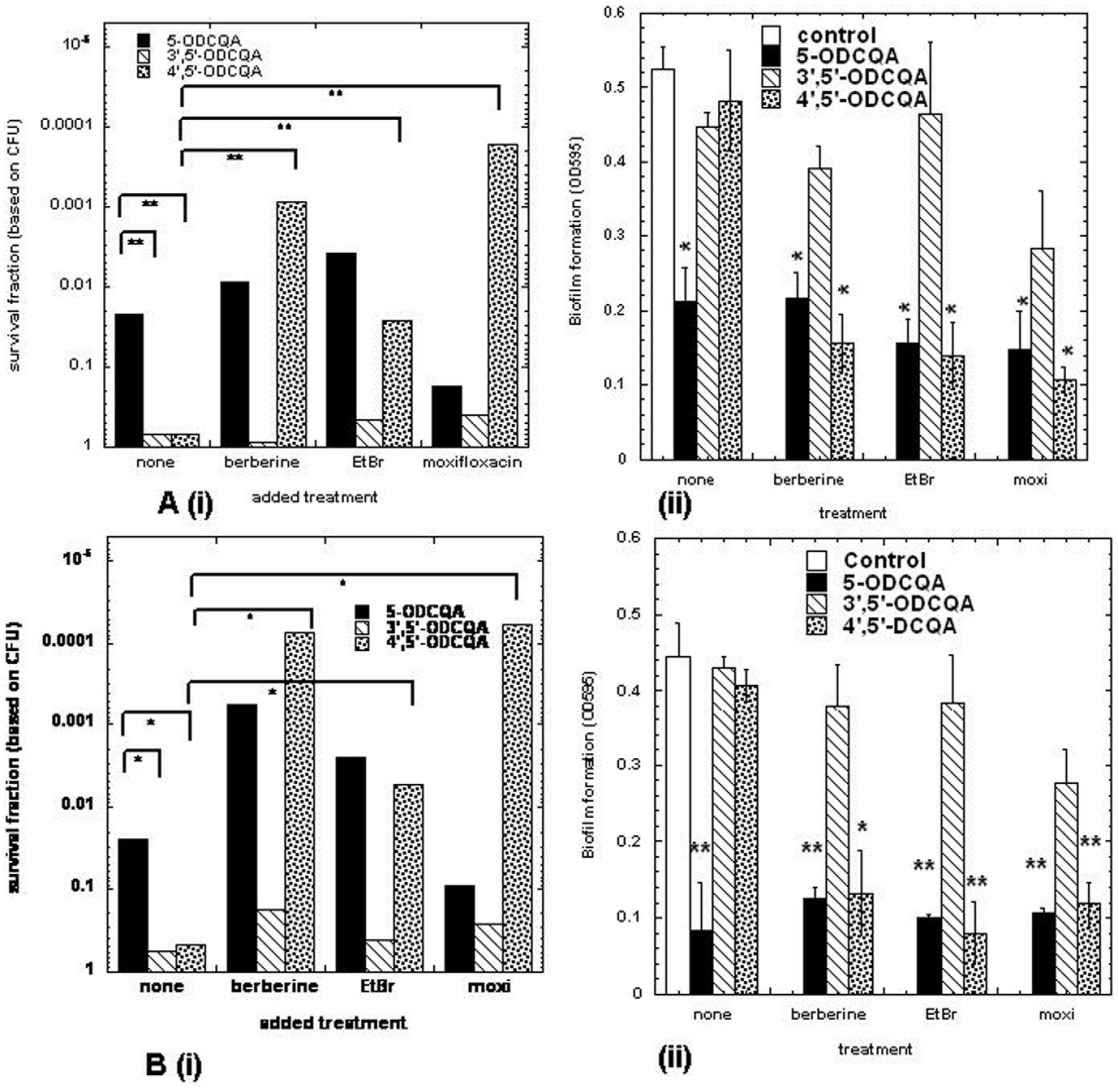
Potentiation of biofilm inactivation by CQAs in combination with model antimicrobials. (A) Biofilms of *S. aureus* 8325-4 were significantly more sensitive to berberine, ethidium bromide and representative fluroquinolones in the presence of the EPI 4′,5′-*O*DCQA. Antimicrobials in sub-lethal/inhibitory concentrations (100 µg/ml for berberine, 10 µg/ml for EtBr and 2 µg/ml for moxifloxacin respectively) were added to 48 hr biofilms that had been grown in media with and without CQAs. After 2 h of exposure to antimicrobials the biofilm density was assessed by determining the number of CFUs per microtiter well (i) and the absorbance of crystal violet (CV)-stained biofilms at 600 nm (ii). In the presence of CQAs (50 µg/ml), (B) Biofilms of *E. faecalis* OGRF1 strain showed large increases in EtBr susceptibility in the presence of 4′5′-*O*DCQA. 48 hr biofilms were exposed to sub-inhibitory concentrations of antimicrobials (50 µg/ml for berberine, 8 µg/ml for EtBr and 1 µg/ml for moxifloxacin respectively) for 2 h after growth in media with and without CQAs. The size of the viable biofilm was found by calculating the number of CFUs per microtiter well (i) and the absorbance of crystal violet (CV)-stained biofilms at 600 nm (ii).for three independent experiments and statistical significance determined by a paired two-tailed *t* test).

In order to determine if members of the natural products of the CQAs family were able to inhibit biofilm formation, alone and in combination with a representative panel of antimicrobials, a standard biofilm assay was conducted. The data are shown for *S. aureus* in **Figure 4A** and for *E. faecalis* in **Figure 4B**. Only 5′-*O*CQA, when added alone, had a significant reduction in biofilm viability in both strains whereas both DCQAs did not. Challenging biofilms with CQA derivatives in combination with sub-inhibitory concentrations of berberine, ethidium bromide, and the fluroquinolone moxifloxacin gave a statistically significant decrease in viability of biofilms in the presence of added 4′,5′-*O*DCQA. The killing effect was increased 2 to 4 logs In the presence of 50 µg/ml 4′,5′-*O*DCQA, it was more pronounced in *E. faecalis* than *S. aureus* for all antimicrobials tested. CQA enhanced the killing effect of the compounds used in a less dramatic fashion when it was added in both biofilm species at the same concentration. By contrast 3′,5′-*O*DCQA had no apparent effect in either microorganism tested, regardless of the antimicrobial employed. Quantitation of biofilm formation and subsequent inhibition with or without treatments was followed by reduction of OD_600_ after Crystal Violet staining and Colony Forming Units (CFU) determinations. Results from the CV stained biofilms were compatible with CFU determinations but were omitted in the presentation for simplicity.

### Molecular modeling studies

**Figure 5:**
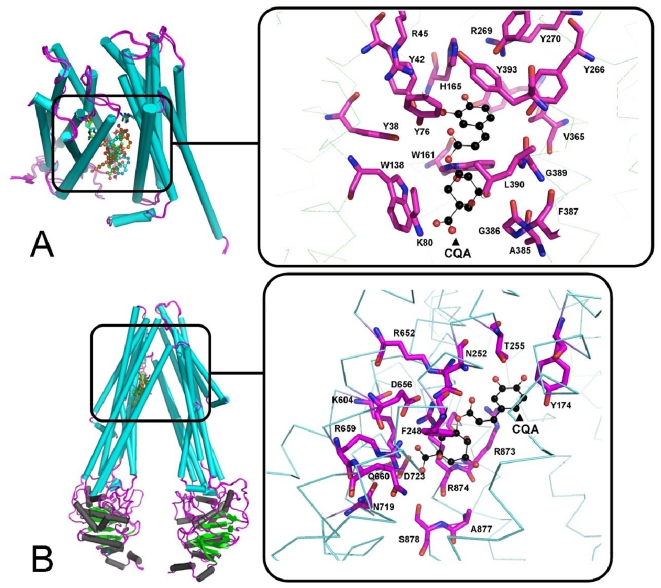
Docking results and interactions within the binding pockets of MES for of all CQAs. All CQAs can successfully enter the binding sites of both efflux systems (A, B). See also Table 3 for the calculated energetics.

In order to gain structural insights into the interaction of the potential EPIs (5′-*O*CQA, 4′,5′-*O*DCQA and 3′,5′-*O*DCQA) with two MES a. an ABC transporter [30] and b. a MFS transporter[31] we used HADDOCK [32]. High quality models could be obtained for all six protein-ligand complexes. Docking results are consistent with our antimicrobial susceptibility tests and the high-throughput competitive efflux assays performed in this study: All CQAs can successfully enter the binding site of both efflux pumps (**Figure 5**) remaining in the binding pockets throughout the simulation runs. In addition, CQAs are predicted to have different interaction energies with each efflux system: Their preferential binding to the MFS transporter is governed by hydrophobic interactions uniformly found in all solutions (**Table 6**). Conversely, when interacting with the ABC transporter, electrostatic forces (ionic interactions and hydrogen bonds) are dominant.

**Table 6:** Energetic contributions and cluster sizes for each docking run.

	Efflux Pump PDB ID^a^	Cluster size^b^	RMSd^d^ (Å)	Haddock Score (kcal/mol)	Van der Waals (kcal/mol)	Electrostatics (kcal/mol)	Desolvation (kcal/mol)	BSA (Å^2^)
5′-*O*CQA	1PW4	198	0.3±0.2	−90.3±7.2	−22.0±0.7	−140.9±18.3	−40.1±10.2	671±38
	2HYD*	146	0.3±0.2	−1.3±4.6	−9.7±3.4	−161.5±24.2	40.6±9.3	498±83
3′,5′-*O*DCQA	1PW4	116	0.3±0.2	−108.2±3.6	−27.7±2.8	−151.5±10.1	−50.2±3.5	825±32
		82	0.5±0.0	−98.1±4.4	−27.1±1.8	−146.7±20.7	−41.7±7.1	823±64
	2HYD*	7	0.3±0.2	0.1±8.0	−22.9±3.5	−182.0±18.6	58.5±2.5	716±57
		94^c^	0.5±0.0	2.8±7.1	−17.9±5.7	−176.8±25.0	55.9±5.4	623±72
4′,5′-*O*DCQA	1PW4	88	0.3±0.2	−108.1±11.0	−38.2±3.0	−137.1±12.2	−42.4±11.5	931±48
		109	0.5±0.1	−103.9±2.9	−32.8±3.4	−169.7±32.1	−37.2±6.3	952±22
	2HYD*	82	0.3±0.2	−8.7±3.9	−20.8±1.5	−185.7±32.5	49.1±5.7	766±70

See text for details and materials and methods for column header definitions.

a 1PW4 corresponds to the PDB ID glycerol-3-phosphate transporter from *Escherichia coli* and 2HYD* to the modeled open conformation of Sav1688 ABC transporter.

b Size of top ranking clusters. Clustering was performed with a 7.5 Å cutoff based on pairwise interface ligand RMSDs, the RMSD of the ligand after fitting on the binding site of the receptor.

c This cluster scored second on the top; the first cluster included only 7 structures and the energetics were very similar (see Table).

d RMSd is the average Root mean square displacement from the top four ranking model of a cluster from its top ranking member, calculated on the backbone atoms of the protein and all heavy atoms of the ligand.

### Interactions of the caffeoylquinic acids with the glycerol-3-phosphate transporter (PDB ID: 1PW4)

5′-*O*CQA exhibits strong binding to the glycerol-3-phosphate transporter (see **Table 6** and **Figure 5, A**). Besides the strong desolvation energy component (-40.1±10.2 kcal/mol), it is also stabilized by increased electrostatics (−140.9±18.3 kcal/mol). For example, two Arg residues (R45 and R269) have been observed to be crucial for the inhibition of the MFS transporter. In our results, their side chains form salt bridges with the oxygen atoms of the 5′-*O*-caffeoylquinic acid (distance <5.5 Å) **(** **Figure 5A**). K80, located at the transporter's active site, forms a salt bridge with the –COOH group of 5′-*O*CQA. Results are also very consistent as nearly all models fall into under one cluster, with an average positional root mean square displacement from the overall lowest-energy structure equal to 0.3±0.1 Å.

For 3′,5′-*O*DCQA, the docking results in two low-energy conformations **(** **Table 6** **)**. Although their interaction energies are quite similar and their location resembles that of the ‘classical inhibitor’, striking differences in their hydrogen bond network are observed (For details, see Figure S3). 4′,5′-*O*DCQA is found in the binding site in an orientation similar to that of 3′,5′-*O*DCQA. However, the significantly larger buried surface area (BSA) and more favorable van der Waals energies are indicative of a tighter fit into the binding site of the MFS efflux system than the other inhibitors; in particular 3′,5′-*O*DCQA does not show such an extent in complementarity.

### Interactions of caffeoylquinic acids with the Sav1688 ABC transporter (PDB ID: 2HYD)

The ABC transporter was modeled in its open conformation (**Figure 5B**) (**Figure S4 and Table S1**), using as template the crystal structure of its close sequential and structural homologue, the P-glycoprotein in its open form (PDB ID: 3G61) [33]. All caffeoylquinic acids successfully entered the active site of the ABC transporter (**Figure 5B**). Besides the favorable Electrostatics (**Figure 5B**), the desolvation penalty is quite large (**Table 6**). Results are rather similar to the ones shown above for the MFS transporters with one major cluster with over 140 members. All other clusters were small in size (<10 structures were included). Binding of this molecule to the ABC transporter is, however, not as energetically favored as to the MFS transporters (for comparison of the interactions, see **Table 6** and **Figure 5A and 5B**). For the other two EPIs, more clusters were obtained. As was already the case with the docking to the MSF transporters, in all solutions, 4′,5′-*O*DCQA occupies a much larger area in the binding site compared to 3′,5′-*O*DCQA; van der Waals interactions of these EPIs also follow the same principle (**Table 6**).

All the above mentioned results can rationalize the strong observed binding of 4′,5′-*O*DCQA and 5′-*O*CQA and the lower efflux pump inhibition of 3′,5′-*O*DCQA. They suggest that, for the MFS efflux systems, all molecules could potentially inhibit the active site; however, 5′-*O*CQA exhibits in both cases a substantially smaller interaction region showing less complementarity. The docking of all CQAs in the binding sites of the ABC transporter, reveals an abundance of charged interactions. 3′,5′-*O*DCQA shows a decreased complementarity within the active site compared to that of 4′,5′-*O*DCQA. Observed interactions with residues within the active sites are *similar* to the ones described in the literature for other EPIs [34], [35].

We, therefore, formulate a hypothesis consistent with the derived energetics, that CQAs show a preferential binding to MFS efflux systems, rather than to ABC transporters. The reported energies provide insights into the interaction of the EPIs with efflux systems and contribute to a working hypothesis regarding the molecular mechanisms behind their observed inhibitory activities. They should, however, be interpreted with caution and only in terms of estimates of potential of binding and not as binding affinities [36]. Studies including Surface Plasmon Resonance or Isothermal Titration Calorimetry to determine K_d_'s or ΔG's will offer further insights in this primary hypothesis.

### Synopsis

An array of natural product screening and synthesis campaigns led to the identification of a variety EPI chemotypes targeting NorA. The list includes catechin gallates [37], the resin glycosides and tetrasaccharide agents of *Ipomoea murucoides* [38], polyacylated oligosaccharides from the medicinal Mexican morning glory species [39], N-caffeoylphenalkylamide derivatives [40], citral derived amides [41] kaempferol glycoside from *Herissantia tiubae* (Malvaceae) [42] and a set of plant-based alkaloids against methicillin-resistant *S. aureus* (MRSA) [43]. A series of plant phenolic compounds have been functioning as ethidium bromide EPIs in *Mycobacterium smegmatis* [44]. A series of synthetic efforts have been concentrated in paroxetine and femoxetine [45], piperine and fluoroquinolone as the basis for designing structural analogues [46], [47], phenothiazines [48] with emphasis on thioridazine and chlorpromazine [49]. All these explorations are focusing in the bioassay driven purification and structural determination of natural EPIs employing an EtBr effux assay. In retrospective this venture offers a rapid methodological alternative to the direct isolation of active compounds as well as attempts to enrich the EPI discovery process with an extensive and rationalized secondary validation of lead chemotypes. The antimicrobial and toxicity profile of caffeoylquinic acids combined with prior art and information regarding their impact in anti-oxidant defenses in mammalian systems suggests a potential similar role in bacteria. The direct antimicrobial activity of CQA implies an array of possibilities including effect in the cell envelope and requires a robust target based validation and investigation.

## Materials and Methods

### Microbial strains and culture conditions

The following bacterial strains were used in this study: *S. aureus* 8325-4 (wild-type); K1758 (8325-4 Δ*norA*); QT1 8325-4 *norR*::*cat* (NorA is overexpressed [50], *S. aureus* Community Associated (CA)-MRSA USA300 (Human isolate/Clinical Microbiology, MGH), *E. faecalis* OGRF1 (Fus^r^ Rif^r^; *p*-Cl-Phe^r^), *E. faecalis* GW4481 (OG1RF*ΔemeA,* [51], *Escherichia coli* K12 (ATCC), prototrophic wild type reference strain *Candida albicans* DAY185 [52]. Bacterial cells were cultured in Mueller–Hinton Broth (MHB) and yeast cells in YPD (unless is otherwise stated). Cell growth was assessed with a spectrophotometer (Shimadzu, Mini 1240) at 600 nm (OD_600_).

### Chemicals

All solvents used in chromatography were of HPLC grade from Lab-Scan Analytical Sciences Ltd. (Dublin Ireland); Formic acid from Merck (Haarlem, The Netherlands) and H_2_O was produced in-house (Milli-Q Water Purification System, Millipore). Acetonitrile-d_3_ (99.8%) was obtained from Cambridge Isotope Laboratories (Apeldoorn, The Netherlands). Antibiotics (erythromycin, tetracycline) and antimicrobials (berberine, ethidium bromide, rhodamine 6G, chlorogenic acid) were purchased from Sigma Chemical Co. (St. Louis, Mo.). Fluroquinolones (norfloxacin, ciprofloxacin, levofloxacin and moxifloxacin) were from the Massachusetts General Hospital Pharmacy (http://www.massgeneral.org/services/pharmacy.aspx) INF_271_ was provided by Chembridge Inc. San Diego, CA) 1′,3′ & 1′,5′-*O*DCQA as well as peracetylderivatives of both DCQAs, were kindly provided by Jiri Slanina (Department of Biochemistry Faculty of Medicine Masaryk University in Brno [53] and 3′,5′-*O*DCQA, 4′,5′-*O*DCQA by Cfm Oskar Tropitzsch e.K. (Marktredwitz, Germany).

### Plant Material & Aqueous infusions

*Artemisia absinthium,* growing wild in Epirus region (Northwestern Greece) was collected in the period from spring to summer. 2 g of dried aerial parts of *A. absinthium,* ground to pass a 0.4 mm sieve, were added to 50 ml of boiling double distilled water (DDW) and stirred for 30 min. The plant residue was then filtered, and the filtrate was lyophilized. The 40 mg of dried extract produced were analyzed for the determination of its main active components. Plant materials and aqueous infusions preparations as well as instrumentation used for the analysis of the constituents are reported in **Text S1**.

### Susceptibility testing

Cells (10^5^/ml) were inoculated into MHB (RPMI for *C. albicans*) and dispensed at 0.2 ml/well in 96-well microtiter plates. Growth inhibition was determined by serial 2-fold dilution of test extract or compounds in 96-well microtiter plates to determine Minimal Inhibitory Concentrations (MICs). In order to identify compounds with EPI activity, extracts and compounds were serially 2-fold diluted in combination with 30 µg/ml berberine for gram-positive bacteria, 10 µg/ml erythromycin for *E. coli*, and 10 µg/ml rhodamine 6G (R6G) for yeast cells. An EPI was defined as a compound that completely prevented cell growth in the presence of sub-inhibitory antibiotic during an 18-hr incubation at 37°C or 24–48 hr at 30°C (*B. cereus, C. albicans*). All tests were done in triplicate by following the Clinical and Laboratory Standards Institute (CLSI, former NCCLS, National Center for Clinical Laboratory Standards) recommendations [54], [55]. Growth was assayed with a microtiter plate reader (Spectramax PLUS384, Molecular Devices) by absorption at 600 nm. The checkerboard tests for DCQAs and antimicrobials were performed as previously described [56]. For each combination experiment (composed of a row or column in the matrix), the Fractional Inhibitory Concentration (FIC) of each agent was calculated: FIC = 1 additive effect; FIC<1, synergy; FIC>1 antagonism.

### Hemolysis assay

The ability of CQAs to hemolyze sheep erythrocytes is based on the protocol of Ciornei *et al*. [57], with the following modifications [58]: sheep erythrocytes (Rockland Immunochemicals) were treated with CQAs (0.5–50 µg/ml) in PBS with DMSO at 2% and the supernatants for 2 h, using Triton X-100 and DMSO as controls, and were monitored on a microtiter plate reader (Spectramax PLUS384, Molecular Devices) at OD_540_.

### Berberine accumulation assays

Determination of berberine uptake was performed as described previously [9], [59]. Cells were cultured with aeration at 37°C to an optical density at 600 nm (OD_600_) of 1.8, pelleted, and washed twice with 20 mM HEPES-NaOH (pH 7.0) buffer. The cells were then resuspended to an OD_600_ of 0.3 in 1 ml of HEPES buffer containing 10 µM glucose followed by incubation at 37 or 30°C for 1 h. The cells were centrifuged, washed, and resuspended at an OD_600_ of 0.15 in HEPES buffer. Assays were performed in 96-well flat-bottom black plates (Costar) in a final volume of 200 µl. Berberine was added at 30 µg/ml, and fluorescence was measured with a Spectramax Geminis spectrofluorometer (Molecular Devices) at a 355-nm excitation wavelength and a 517-nm emission wavelength.

### Berberine competitive efflux assay

Those were performed as described [59]. *S. aureus* and *E. faecalis* cells were cultured with aeration and mild agitation until they reached the late log phase (OD_600_ between 0.9 and 1), pelleted by centrifugation (2 min, 12,000 rpm), and then washed and resuspended in 25 mM PBS (pH 7.4) containing 0.05 g L^−1^ MgSO_4_, 7 g L^−1^ K_2_HPO_4_, 0.5 g L^−1^ sodium citrate·3H_2_O, 1 g L^−1^ (NH_4_)_2_SO_4_, 0.01 mg L^−1^ folic acid, 0.05 mg L^−1^ pyridoxine hydrochloride, 0.025 mg L^−1^ riboflavin, 0.01 mg L^−1^ biotin, 0.025 mg L^−1^ thiamine, 0.025 mg L^−1^ nicotinic acid, 0.025 mg l^−1^ calcium pantothenate, 0.5 µg L^−1^ vitamin B12, 0.025 mg L^−1^ *p*-aminobenzoic acid, 0.025 mg L^−1^ thiotic acid, and 4.5 mg L^−1^ monopotassium phosphate. Cells were then resuspended to an OD_600_ of 0.8 in buffer with 10 mM glucose. Cells were then loaded with either 30 µg/ml berberine and 10 µg/ml 4′,5′-*O*DCQA and incubated at 37°C with aeration for 20 min. Cells were then centrifuged in a 4°C cold room, washed in ice-cold PBS, and added at an OD_600_ of 0.3 to a chilled 96-well flat-bottom white microtiter plate (Costar) containing ice-cold 25 mM PBS and 10 mM glucose in a final volume of 200 µL. Fluorescence was measured with a Spectramax PLUS384 GeminiXS spectrofluorometer (Molecular Devices) at a 355-nm excitation wavelength and a 517-nm emission wavelength.

### Biofilm formation & Quantitation

Biofilm formation is assayed by the ability of cells to adhere on polystyrene surface of 24-well microtiter plates [60]. The indicated medium is inoculated from a 1∶100 dilution from an overnight BHI culture. Plates are incubated at 37°C for 48 hrs. Compounds are added at the appropriate combinations and concentrations followed by additional overnight incubation at 37°C. Two identical microtiter plates were used for each independent experiment. For the first plate crystal violet (CV) solution was added to each well, the plates were incubated at room temperature, rinsed thoroughly with water and dissolved. CV-stained biofilms were solubilized in ethanol, and samples were transferred to a new polystyrene microtiter dish, and the absorbance was determined with a plate reader at 600 nm. For the second plate cells are dissolved followed by CFU determinations on BHI agar plates.

### Statistics

For the toxicity experiments studies values are means of three separate experiments and bars are SEM. For biofilm inactivation studies, between means were tested for significance by a paired two-tailed *t* test The significance level was set at p<0.05.

### Structure-activity relationships and comparative protein-ligand docking studies

Two efflux pump crystal structures [34], [35] were selected to perform comparative docking of caffeoylquinic acids to their binding sites: the glycerol-3-phosphate transporter from *Escherichia coli* [35] (PDB ID: 1PW4) was the representative structure from the MFS, and Sav1866 from *Staphylococcus aureus* [34], [35] (PDB ID: 2HYD), is an ABC transporter of Gram-positive bacteria. 2HYD was crystallized in the closed conformation. Since 2HYD shares very high structural similarity with P-glycoprotein [34], [61], the open conformation was generated by flexible structural alignment with FATCAT (http://fatcat.burnham.org/) against the corresponding P-glycoprotein crystal structure [61] (PDB ID: 3G61). This approach concluded in a good quality model (see **Figure S3 and Table S1**).

For the docking trials, HADDOCK version 2.1 [62] was used. HADDOCK is a highly successful modeling approach that makes use of biochemical and/or biophysical interaction data such as chemical shift perturbation data, mutagenesis data, or bioinformatic predictions and thus, incorporates structural knowledge of the target to drive the docking procedure. The docking was performed with default parameters using the web server version of HADDOCK [63]. All calculations were performed with CNS1.2 [64]. Non-bonded interactions were calculated with the OPLS force field [65] using a cutoff of 8.5 Å. The electrostatic potential (E_elec_) was calculated by using a shift function, while a switching function (between 6.5 and 8.5 Å) was used to define the Van der Waals potential (E_vdw_). The HADDOCK score is used to rank the generated poses. It is a weighted sum of intermolecular electrostatic (E_Elec_), van der Waals (E_vdW_), desolvation (ΔG_solv_) and ambiguous interaction restraint (AIR) energies with weight factors of 0.2, 1.0, 1.0 and 0.1, respectively.

The ambiguous interaction restraints to drive the docking were defined as follows. The ligand was treated as an active residue for all stages of the docking, while the residues within the protein binding site were only defined as active for the rigid-body docking stage and considered as passive the subsequent semi-flexible refinement stage. This strategy allows to effectively pulling the ligand within the binding site during rigid-body docking while allowing a more thorough exploration of the binding pocket during the refinement stage. Residues considered active for 1PW4 were 38, 42, 45, 46, 134, 165, 166, 266, 269, 270, 299, 362 and 393, whereas for 2HYD*, the active residues were 26, 252, 255, 288, 291, 295, 604, 608, 611, 612, 614, 648, 722, 837, 866, 869, 870, 873, 874 and 896. Passive residues were defined automatically via the web-server, as those surrounding the active ones.

## Supporting Information

Figure S1 **400 MHz 1H-NMR spectrum of 1, in CD3CN.** The spin system corresponding to 5′-*O*CQA is revealed. The H-2/and H-6/signals of the quinic moiety are overlapped by the CD_3_CN-H_2_O residual signals and, therefore, they are eliminated.
(TIFF)

Figure S2 **400 MHz 1H-NMR spectrum of 2A and 2B, in CD3CN.** The spin system of 3′,5′-*O*DCQA (2A) is mainly indicated while that one of 4′,5′-*O*DCQA (2B) is suggested in italics. The signals of the aromatic protons of 2B are greatly overlapped by those of the 2A isomer and, thus, are not indicated in the spectrum.
(TIFF)

Figure S3 **View of protein-ligand hydrogen bonds in the top two clusters of the modelled complex of 3′,5′-** ***O*** **DCQA with 1PW4: (A) top ranking cluster, (B) second, most populated cluster.** The different orientations of the 3′,5′-*O*DCQA is reflected in the differences in the hydrogen bonding pattern. For example, 3′,5′-*O*DCQA, show a different pattern of hydrogen bonds of the carbonyl groups, interacting in one case with the side chains of Y38, Y42 and W261 whereas in the second cluster the observed hydrogen bonds are different and fewer in number This figure was generated with PyMol (www.pymol.org).
(TIFF)

Figure S4 **Ramachandran plot of the model of Sav1688 in the open conformation.** The plot is based on an analysis of 118 structures of resolution of at least 2.0 Å and an R-factor no greater than 20%. A good quality model would be expected to have over 90% in the most favoured regions. Plot statistics: Residues in most favoured regions [A,B,L], 1001 (95.5%). Residues in additional allowed regions, [A,B,L,P], 43 (4.1%). Residues in generously allowed regions, [∼A, ∼B, ∼L, ∼P], 2 (0.2%). Residues in disallowed regions, 2 (0.2%). Number of non-glycine and non-proline residues, 1048 (in total: 100.0%). Number of end-residues (excl. Gly and Pro), 4. Number of glycine residues (shown as triangles), 74. Number of proline residues, 30. Total number of residues: 1156.
(TIFF)

Table S1 **PSVS (** **http://psvs-1_4-dev.nesg.org/** ) validation statistics of the modelled open form (2HYD*) and, for comparison, of the crystal structure 2HYD.
(TIFF)

Text S1 **Plant materials and aqueous infusions preparations as well as instrumentation used for the analysis of the constituents of** ***Artemisia absinthium*** **extracts.**
(DOC)

## References

[1] Paulsen IT, Chen J, Nelson KE, Saier MHJ, Lewis K (2002). Comparative genomics of microbial drug efflux systems.. Microbial Multidrug Efflux.

[2] Piddock L (2006). Clinically relevant chromosomally encoded multidrug resistance efflux pumps in bacteria.. Clin Microbiol Rev.

[3] Poole K (2005). Efflux-mediated antimicrobial resistance.. J Antimicrob Chemother.

[4] Piddock LJV (2006). Multidrug-resistance efflux pumps - Not just for resistance.. Nat Rev Microbiol.

[5] Lomovskaya O, Watkins W (2001). Inhibition of efflux pumps as a novel approach to combat drug resistance in bacteria.. J Mol Microbiol Biotechnol.

[6] Lomovskaya O, Zgurskaya HI, Totrov M, Watkins WJ (2007). Waltzing transporters and ‘the dance macabre' between humans and bacteria.. Nat Rev Drug Discov.

[7] Lewis K, Ausubel FM (2006). Prospects for plant-derived antibacterials.. Nat Biotechnol.

[8] Tegos G, Rai M, Carpinella CM (2006). Substrates and Inhibitors of microbial efflux pumps; Redefine the Role of Plant Antimicrobilas Naturally occurring bioactive compounds: a new and safe alternative for control of pests and microbial diseases;.

[9] Tegos G, Stermitz F, Lomovskaya O, Lewis K (2002). Multidrug pump inhibitors uncover remarkable activity of plant antimicrobials.. Antimicrob Agents Che.

[10] Stermitz FR, Lorenz P, Tawara JN, Zenewicz LA, Lewis K (2000). Synergy in a medicinal plant: antimicrobial action of berberine potentiated by 5′-methoxyhydnocarpin, a multidrug pump inhibitor.. Proc Natl Acad Sci U S A.

[11] Belofsky G, Carreno R, Lewis K, Ball A, Casadei G (2006). Metabolites of the "smoke tree", Dalea spinosa, potentiate antibiotic activity against multidrug-resistant Staphylococcus aureus.. J Nat Prod.

[12] Belofsky G, Percivil D, Lewis K, Tegos G, Ekart J (2004). Phenolic Metabolites of *Dalea versicolor* that Enhance Antibiotic Activity Against Multi-Drug Resistant Bacteria.. J Nat Prod.

[13] Morel C, Stermitz FR, Tegos G, Lewis K (2003). Isoflavones as potentiators of antibacterial activity.. J Agric Food Chem.

[14] Stavri M, Piddock LJV, Gibbons S (2007). Bacterial efflux pump inhibitors from natural sources.. J Antimicr Chemother.

[15] Stermitz F, Cashman KK, Halligan KM, Morel C, Tegos G (2003). Polyacylated neohesperidosides from Geranium caespitosum Bacterial multidrug resistance pump inhibitors.. Bioorg Med Chem Lett.

[16] Stermitz F, Scriven L, Tegos G, Lewis K (2002). Two novel flavones from Artemisia annua potentiate the action of berberine against Staphylococcus aureus.. Planta Med.

[17] Juteau F, Jerkovic I, Masotti V, Milos M, Mastelic J (2003). Composition and Antimicrobial Activity of the Essential Oil of Artemisia absinthium from Croatia and France.. Planta Med.

[18] Reinke R, King PJ, Victoria GJ, McDougall RB, Ma G (2002). Dicaffeoyltartaric Acid Analogues Inhibit Human Immunodeficiency Virus Type 1 (HIV-1) Integrase and HIV-1 Replication at Nontoxic Concentrations.. J Med Chem.

[19] Hishamoto M, Kikuzaki H, Ohigashi HN (2003). Antioxidant Compounds from the Leaves of Peucedanum japonicum Thunb.. J Agric Food Chem.

[20] Azuma K, Nakayama M, Koshioka M, Ippoushi K, Yamaguchi Y (1999). Phenolic Antioxidants from the Leaves of Corchorus olitorius L.. J Agric Food Chem.

[21] Ohnishi M, Morishita H, Iwahashi H, Toda S, Shirataki Y (1994). Inhibitory effects of chlorogenic acids on linoleic acid peroxidation and haemolysis.. Phytochemistry.

[22] Tsuchiya T, Suzuki O, Igarashi K (1996). Protective effects of chlorogenic acid on paraquat-induced oxidative stress in rats.. Biosci Biotechnol Biochem.

[23] Exarchou V, Godejohann M, van Beek T, Gerothanassis I, Vervoort J (2003). LC-UV-Solid-Phase Extraction-NMR-MS Combined with a Cryogenic Flow Probe and Its Application to the Identification of Compounds Present in Greek Oregano.. Anal Chem.

[24] Clifford MN, Johnston KL, Knight S, Kuhnert N (2003). Hierarchical Scheme for LC-MSn Identification of Chlorogenic Acids.. J Agric Food Chem.

[25] Bandyopadhyay G, Biswas T, Roy KC, Mandal S, Mandal C (2004). Chlorogenic acid inhibits Bcr-Abl tyrosine kinase and triggers p38 mitogen-activated protein kinase-dependent apoptosis in chronic myelogenous leukemic cells.. Blood.

[26] Singh N, Rajini PS (2008). Antioxidant-mediated protective effect of potato peel extract in erythrocytes against oxidative damage.. Chem-Biol Interact.

[27] Kaatz GW, Seo SM, O'Brien L, Wahiduzzaman M, Foster TJ (2000). Evidence for the existence of a multidrug efflux transporter distinct from NorA in Staphylococcus aureus.. Antimicrob Agents Chemother.

[28] Samosorn S, Bremner JB, Ball A, Lewis K (2006). Synthesis of functionalised 2-aryl-5-nitro-1H-indoles and their activity as bacterial NorA efflux pump inhibitors.. Bioorg Med Chem.

[29] Markham P, Westhaus E, Klyachko K, Johnson ME, Neyfakh AA (1999). Multiple novel inhibitors of the NorA multidrug transporter of Staphylococcus aureus.. Antimicrob Agents Chemother.

[30] Dawson R, Locher KP (2006). Structure of a bacterial multidrug ABC transporter.. Nature.

[31] Huang Y, Lemieux MJ, Song J, Auer M, Wang DN (2003). Structure and mechanism of the glycerol-3-phosphate transporter from Escherichia coli.. Science.

[32] de Vries S, van Dijk M, Bonvin AM (2010). The HADDOCK web server for data-driven biomolecular docking.. http://haddock.chem.uu.nl/services/HADDOCK.

[33] Huang Y, Lemieux MJ, Song J, Auer M, Wang DN (2003). Structure and mechanism of the glycerol-3-phosphate transporter from Escherichia coli.. Science.

[34] Dawson RJP, Locher KP (2006). Structure of a bacterial multidrug ABC transporter.. Nature.

[35] Huang XY, Huang XJ, Cui HR, Zhang JH, Chen SM (2003). Present status of antibiotic resistance of clinical isolates from 1998 to 2002 and the rational use of antibiotics.. Chinese J Antibiot.

[36] Kastritis PL, Bonvin AM (2010). Are scoring functions in protein-protein docking ready to predict interactomes? Clues from a novel binding affinity benchmark.. J Proteom Res.

[37] Gibbons S, Moser E, Kaatz GW (2004). Catechin gallates inhibit multidrug resistance (MDR) in Staphylococcus aureus.. Planta Med.

[38] Chérigo L, Pereda-Miranda R, Fragoso-Serrano M, Jacobo-Herrera N, Kaatz GW (2008). Inhibitors of bacterial multidrug efflux pumps from the resin glycosides of Ipomoea murucoides.. J Nat Prod.

[39] Pereda-Miranda R, Kaatz GW, Gibbons S (2006). Polyacylated oligosaccharides from medicinal Mexican morning glory species as antibacterials and inhibitors of multidrug resistance in Staphylococcus aureus.. J Nat Prod.

[40] Michalet S, Cartier G, David B, Mariotte AM, Dijoux-franca MG (2007). N-caffeoylphenalkylamide derivatives as bacterial efflux pump inhibitors.. Bioorg Med Chem Lett.

[41] Thota N, Koul S, Reddy MV, Sangwan PL, Khan IA (2008). Citral derived amides as potent bacterial NorA efflux pump inhibitors.. Bioorg Med Chem.

[42] Falcão-Silva V, Silva DA, Souza Mde F, Siqueira-Junior JP (2009). Modulation of drug resistance in Staphylococcus aureus by a kaempferol glycoside from Herissantia tiubae (Malvaceae).. Phytother Res.

[43] Mohtar M, Johari SA, Li AR, Isa MM, Mustafa S (2009). Inhibitory and resistance-modifying potential of plant-based alkaloids against methicillin-resistant Staphylococcus aureus (MRSA).. Curr Microbiol.

[44] Lechner D, Gibbons S, Bucar F (2007). Plant phenolic compounds as ethidium bromide efflux inhibitors in Mycobacterium smegmatis.. J Antimicrob Chemother.

[45] Wei P, Kaatz GW, Kerns RJ (2004). Structural differences between paroxetine and femoxetine responsible for differential inhibition of Staphylococcus aureus efflux pumps.. Bioorg Med Chem Lett.

[46] Sangwan P, Koul JL, Koul S, Reddy MV, Thota N (2008). Piperine analogs as potent Staphylococcus aureus NorA efflux pump inhibitors.. Bioorg Med Chem.

[47] German N, Wei P, Kaatz GW, Kerns RJ (2008). Synthesis and evaluation of fluoroquinolone derivatives as substrate-based inhibitors of bacterial efflux pumps.. Eur J Med Chem.

[48] Sabatini S, Kaatz GW, Rossolini GM, Brandini D, Fravolini A (2008). From phenothiazine to 3-phenyl-1,4-benzothiazine derivatives as inhibitors of the Staphylococcus aureus NorA multidrug efflux pump.. J Med Chem.

[49] Rodrigues L, Wagner D, Viveiros M, Sampaio D, Couto I (2008). Thioridazine and chlorpromazine inhibition of ethidium bromide efflux in Mycobacterium avium and Mycobacterium smegmatis.. J Antimicrob Chemother.

[50] Truong-Bolduc QC, Zhang X, Hooper DC (2003). Characterization of NorR protein, a multifunctional regulator of norA expression in Staphylococcus aureus.. J Bacteriol.

[51] Jonas BM, Murray BE, Weinstock GM (2001). Characterization of emeA, a norA homolog and multidrug resistance efflux pump, in Enterococcus faecalis.. Antimicrobial Agents and Chemotherapy.

[52] Nobile CJ, Andes DR, Nett JE, Smith FJ, Yue F (2006). Critical role of Bcr1-dependent adhesins in C. albicans biofilm formation in vitro and in vivo.. PLoS pathogens.

[53] Schram K, Miketova P, Slanina J, Humpa O, Taborska E (2004). Mass spectrometry of 1,3- and 1,5-dicaffeoylquinic acids.. Journal of Mass Spectrometry.

[54] Clinical and Laboratory Standards Institute (2008). Reference Method for Broth Dilution Antifungal Susceptibility Testing of Yeasts..

[55] Clinical and Laboratory Standards Institute (2008). Reference Method for Broth Dilution Antimicrobial Susceptibility Testing..

[56] Eliopoulos GM, Moellering RC, Lorian V (1991). Antibiotics in laboratory medicine.. Antibiotics in laboratory medicine.

[57] Ciornei CD, Sigurdardóttir T, Schmidtchen A, Bodelsson M (2005). Antimicrobial and chemoattractant activity, lipopolysaccharide neutralization, cytotoxicity, and inhibition by serum of analogs of human cathelicidin LL-37 Antimicrob Agents Chemother.

[58] Breger J, Fuchs BB, Aperis G, Moy TI, Ausubel FM (2007). Antifungal chemical compounds identified using a C. elegans pathogenicity assay.. PLoS Pathog.

[59] Ball AR, Casadei G, Samosorn S, Bremner JB, Ausubel FM (2006). Conjugating berberine to a multidrug efflux pump inhibitor creates an effective antimicrobial.. ACS chemical biology.

[60] O'Toole GA, Kolter R (1998). Initiation of biofilm formation in Pseudomonas fluorescens WCS365 proceeds via multiple, convergent signalling pathways: a genetic analysis.. Mol Microbiol.

[61] Aller SG, Yu J, Ward A, Weng Y, Chittaboina S (2009). Structure of P-glycoprotein reveals a molecular basis for poly-specific drug binding.. Science.

[62] Dominguez C, Boelens R, Bonvin AMJJ (2003). HADDOCK: A protein-protein docking approach based on biochemical or biophysical information.. Journal of the American Chemical Society.

[63] de Vries SJ, van Dijk M, Bonvin AM (2010). The HADDOCK web server for data-driven biomolecular docking.. Nature Protocols.

[64] Brunger AT, Adams PD, Clore GM, Delano WL, Gros P (1998). Crystallography & NMR system: A new software suite for macromolecular structure determination.. Acta Crystallogr D.

[65] Jorgensen WL, Tirado-Rives J (1988). The OPLS potential functions for proteins. Energy minimizations for crystals of cyclic peptides and crambin.. Journal of the American Chemical Society.

