# Supplementary figures and images for: Antimicrobial and Efflux Pump Inhibitory Activity of Caffeoylquinic Acids from Artemisia absinthium against Gram-Positive Pathogenic Bacteria
Source: PLoS One. 2011 Apr 4;6(4):e18127. doi: 10.1371/journal.pone.0018127 (PMC3070693; doi:10.1371/journal.pone.0018127)

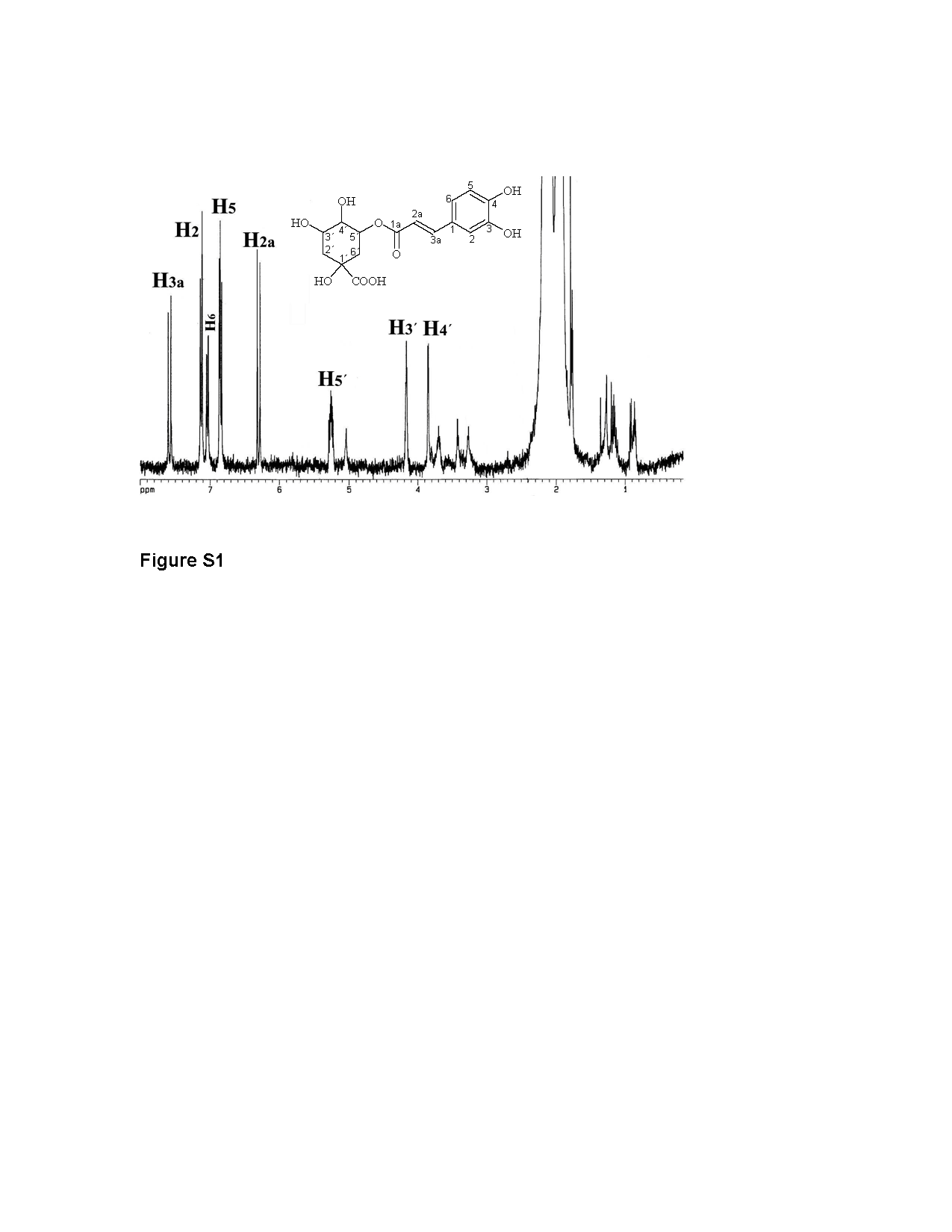

Supplement: Figure S1 — 400 MHz 1H-NMR spectrum of 1, in CD3CN. The spin system corresponding to 5′-OCQA is revealed. The H-2/and H-6/signals of the quinic moiety are overlapped by the CD3CN-H2O residual signals and, therefore, they are eliminated. (TIFF) [file pone.0018127.s001.tif]

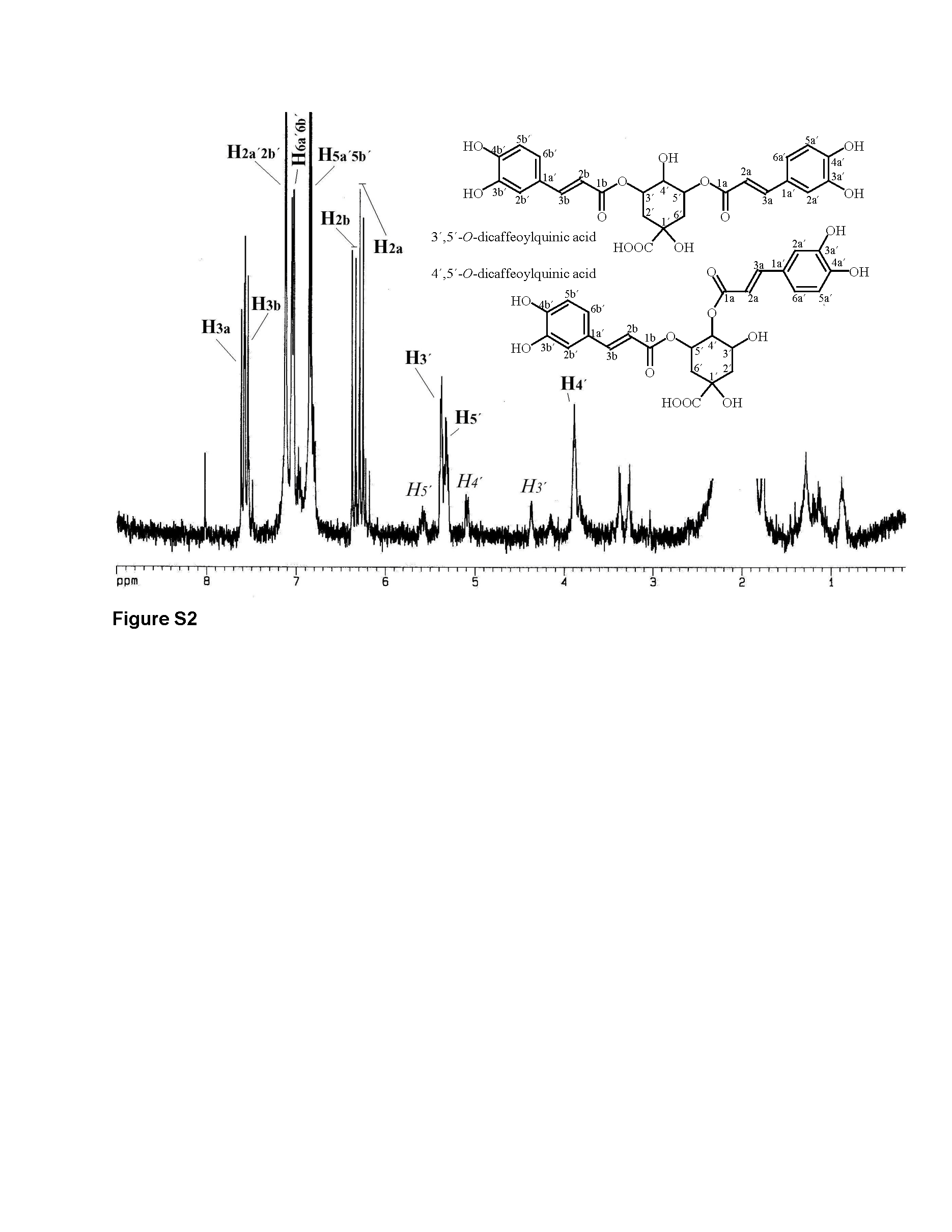

Supplement: Figure S2 — 400 MHz 1H-NMR spectrum of 2A and 2B, in CD3CN. The spin system of 3′,5′-ODCQA (2A) is mainly indicated while that one of 4′,5′-ODCQA (2B) is suggested in italics. The signals of the aromatic protons of 2B are greatly overlapped by those of the 2A isomer and, thus, are not indicated in the spectrum. (TIFF) [file pone.0018127.s002.tif]

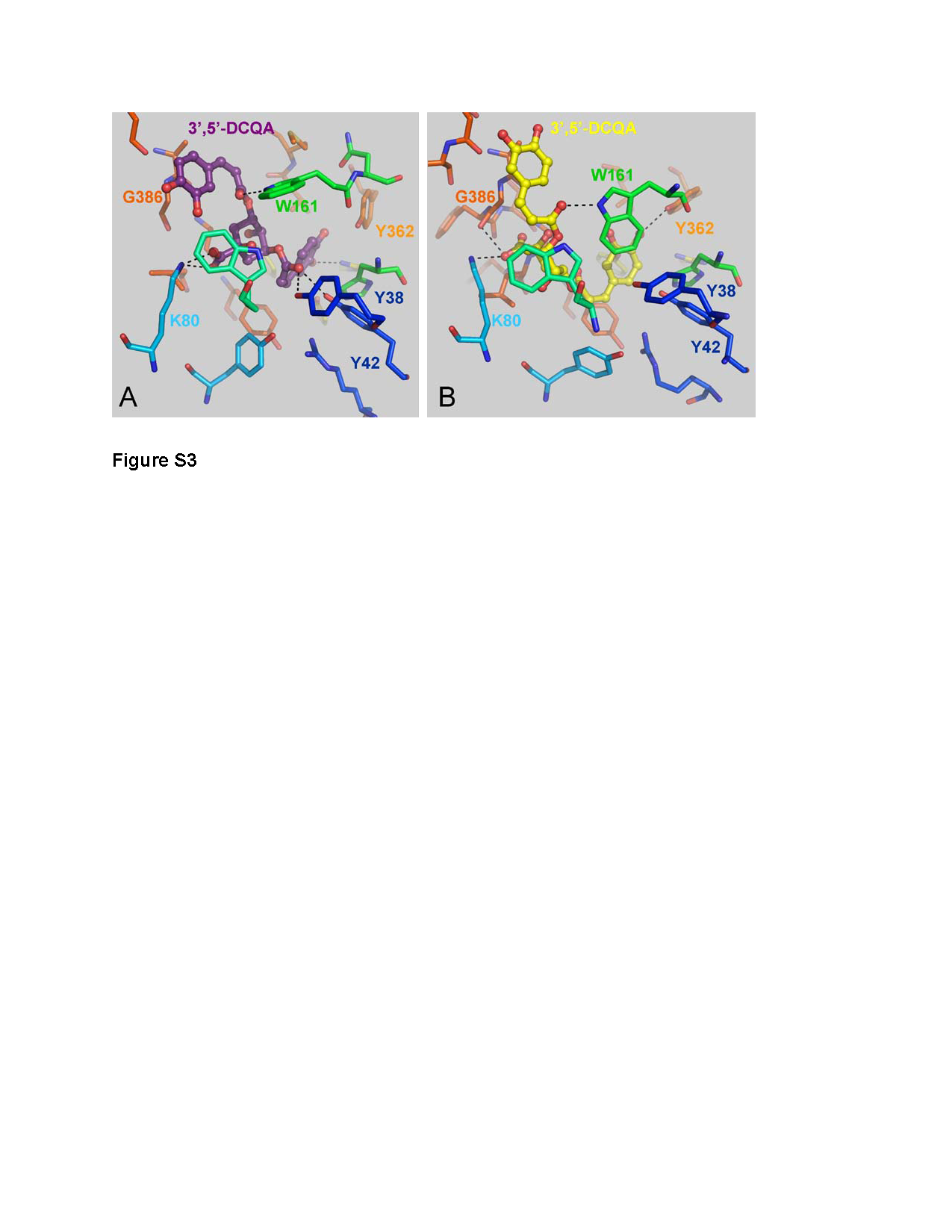

Supplement: Figure S3 — View of protein-ligand hydrogen bonds in the top two clusters of the modelled complex of 3′,5′- O DCQA with 1PW4: (A) top ranking cluster, (B) second, most populated cluster. The different orientations of the 3′,5′-ODCQA is reflected in the differences in the hydrogen bonding pattern. For example, 3′,5′-ODCQA, show a different pattern of hydrogen bonds of the carbonyl groups, interacting in one case with the side chains of Y38, Y42 and W261 whereas in the second cluster the observed hydrogen bonds are different and fewer in number This figure was generated with PyMol (www.pymol.org). (TIFF) [file pone.0018127.s003.tif]

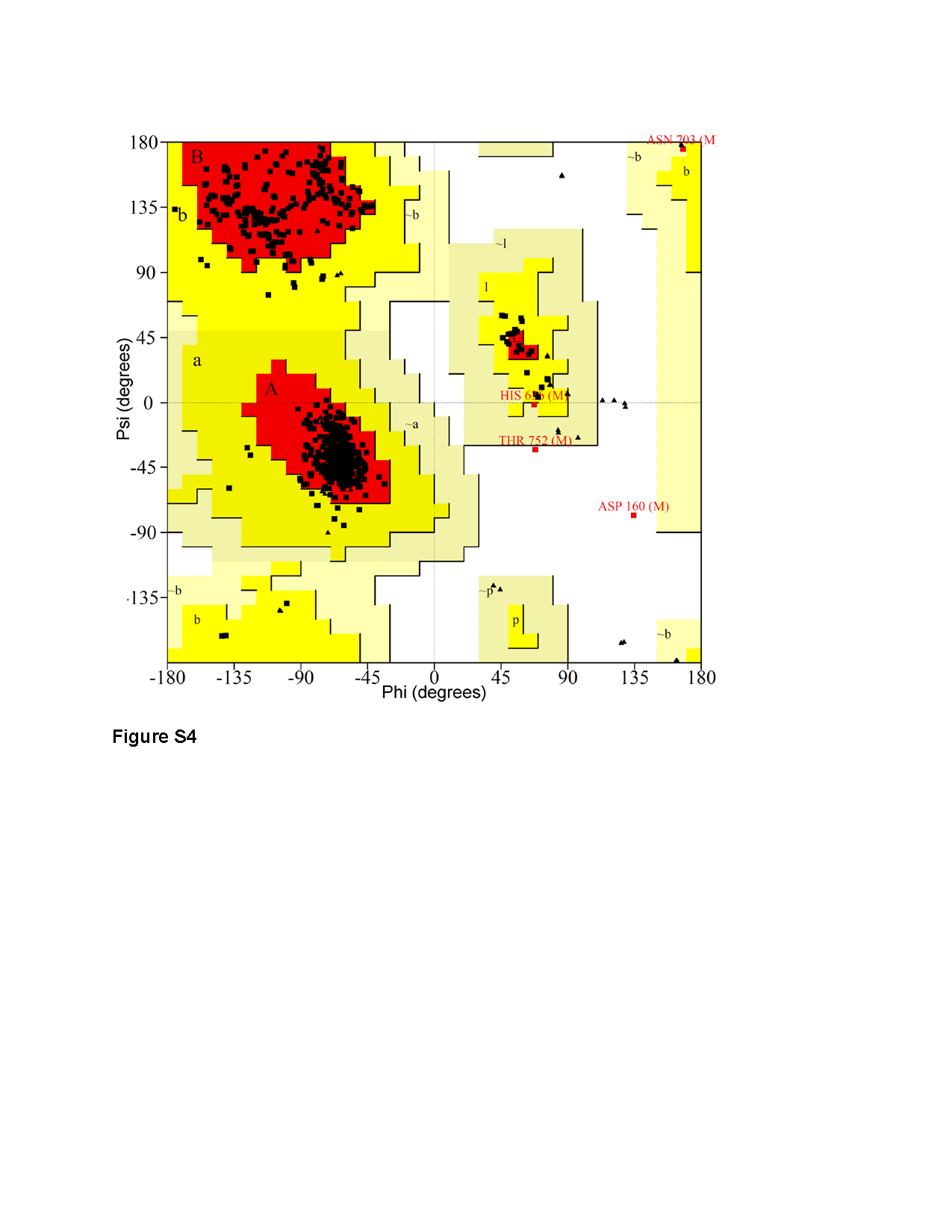

Supplement: Figure S4 — Ramachandran plot of the model of Sav1688 in the open conformation. The plot is based on an analysis of 118 structures of resolution of at least 2.0 Å and an R-factor no greater than 20%. A good quality model would be expected to have over 90% in the most favoured regions. Plot statistics: Residues in most favoured regions [A,B,L], 1001 (95.5%). Residues in additional allowed regions, [A,B,L,P], 43 (4.1%). Residues in generously allowed regions, [∼A, ∼B, ∼L, ∼P], 2 (0.2%). Residues in disallowed regions, 2 (0.2%). Number of non-glycine and non-proline residues, 1048 (in total: 100.0%). Number of end-residues (excl. Gly and Pro), 4. Number of glycine residues (shown as triangles), 74. Number of proline residues, 30. Total number of residues: 1156. (TIFF) [file pone.0018127.s004.tif]

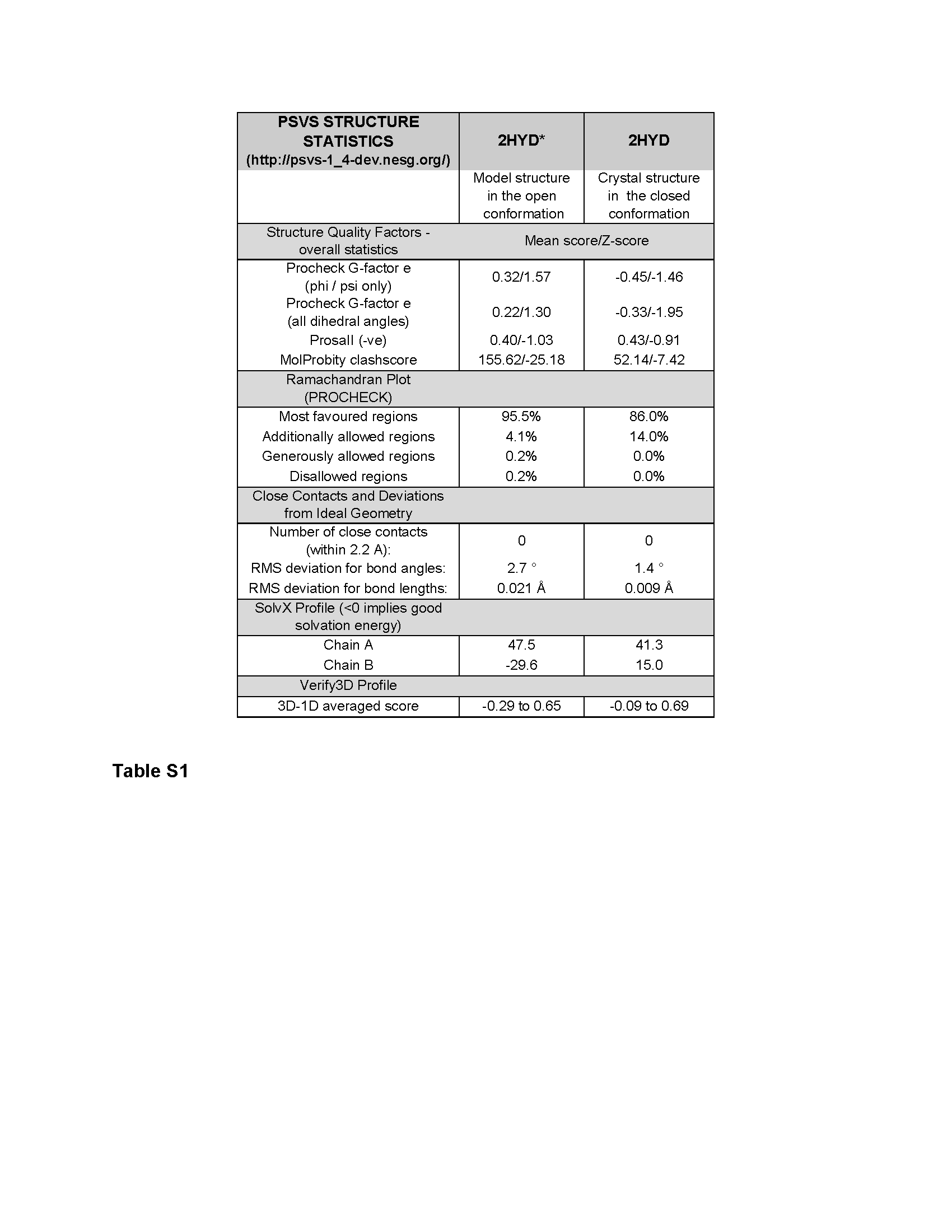

Supplement: Table S1 — PSVS ( http://psvs-1_4-dev.nesg.org/ ) validation statistics of the modelled open form (2HYD*) and, for comparison, of the crystal structure 2HYD. (TIFF) [file pone.0018127.s005.tif]
