# Supplementary material for: Antimicrobial and Efflux Pump Inhibitory Activity of Caffeoylquinic Acids from Artemisia absinthium against Gram-Positive Pathogenic Bacteria
Source: PLoS One. 2011 Apr 4;6(4):e18127. doi: 10.1371/journal.pone.0018127 (PMC3070693; doi:10.1371/journal.pone.0018127)
Supplement: Text S1 — Plant materials and aqueous infusions preparations as well as instrumentation used for the analysis of the constituents of Artemisia absinthium extracts. (DOC) [file pone.0018127.s006.doc]

**Text S1**

**Plant Material & Aqueous infusions.** *Artemisia absinthium,* growing wild in Epirus region (Northwestern Greece) was collected in the period from spring to summer. 2 g of dried aerial parts of *Artemisia absinthium,* ground to pass a 0.4 mm sieve, were added to 50ml of boiling double distilled water DDW and stirred for 30 min. The plant residue was then filtered and the filtrate was lyophilized. The 40 mg of dried extract produced were analyzed for the determination of its main active components.

**Instrumentation.** The LC/MS analyses was performed with a TSP P4000 Quaternary pump coupled with a TSP AS series autosampler connected with a 150 mm x 2.1 mm i.d., 5 μm, Altima C-18 column from Altech (Breda, The Netherlands). The effluents were directed to a double wavelength TSP UV2000 detector on-line with a Finnigan MAT LCQ ion trap mass spectrometer (Waters Integrity System, Milford, MA). The flow rate was 0.2 mL/min, and the injection volume was 10 μL. Gradient elution was performed using solvent A 0.1% v/v formic acid in water and solvent B acetonitrile with the following linear gradient combination: at 0 min, 10% B; at 25 min, 70% B; at 29 min, 70% B; at 33 min, 100% B; at 37 min 100% B; at 41 min, 10% B; followed by a 5 min conditioning step. The ESI interface was used in the negative ionisation mode in 200^o^C with –4.5 kV source voltage. MS^2^ spectra were aquired with a 25% collision energy.

LC-UV-SPE-NMR analyses were carried out on a chromatographic separation system consisting of a Bruker LC22 solvent delivery pump and a Bruker Diode Array UV detector (Bruker BioSpin, Rheinstetten, Germany). The sample was injected using a Rheodyne model 7725i injection valve equipped with a 100 μL injection loop (Cotati, USA). The Bruker/Spark Prospekt 2 Solid Phase Extraction Unit (Bruker BioSpin, Rheinstetten, Germany & Spark, Emmen, the Netherlands) was used to automatically trap the chromatographic peaks on Hysphere GP cartridges (2 mm, 10-12 μm) after post column addition of water using a Knauer K100 HPLC pump (Berlin, Germany). The trapped peaks were first dried with dry nitrogen gas and eluted with deuterated acetonitrile into a DPX-400 NMR spectrometer equipped with a 4 mm LC SEI 13C 1H probe head with an active volume of 120 μL from Bruker BioSpin (Rheinstetten, Germany).

The chromatographic separation was carried out on a 150 mm x 4.6 mm i.d., 5 μm, Altima C-18 column from Altech (Breda, The Netherlands). The flow rate was 0.6 mL/min, and the injection volume was 20 μL. Gradient elution was performed using the conditions of LC-MS analysis. During the chromatographic separation, water with 0.1% v/v formic acid was added to the eluent leaving the column with a second HPLC pump (make-up pump), in order to to provide proper retention of the peaks under study on the SPE cartridges of the system. After the chromatographic separation the SPE cartridges were dried with nitrogen gas to remove the residual solvents and subsequently, the analytes were transferred to the NMR probe head with pure acetonitrile-d_3_.

The second and third main components of the extract were co-eluted from the chromatographic column having a molecular ion of m/z = 515 being in fact two dicaffeoylquinic acids isomers. Evaluation of the respective chromatograms and ^1^HNMR spectra revealed that the two isomers exist in an analogy 3:1. MS^2^ analysis of the two isomers gives out that 2A is 3´,5´-*ODCQA* and the 2B is 4´,5´-*O*DCQA acid [24]. Spectral data of the three components are given below. The H-2´ and H-6´ of the quinic moiety are overlapped by the solvent signal as shown in figures S1 and S2. Since compounds 2A and 2B were collected in the same SPE cartridges and the resolution for the ^1^HNMR spectra of 4´,5´-*O*DCQA acid was not the optimum.

**5´-*O*-caffeoylquinic acid (1):** UVmax, 326 nm, MS (ESI) m/z= 353.0, (MS^2^: 191.2), ^1^HNMR (d_3_-Acetonitrile), *δ* (ppm), 3.71 (1H, dd, H-4´), 5.29 (1H, m, H-5´), 4.18 (1H, m, H-3´a), 6.33 (1H, d, H-2a, *J*=15.99), 6.87 (1H, d, H-5΄, *J*=8.11), 7.06 (1H, d, H-6΄, *J*=8.25), 7.14 (1H, s, H-2΄), 7.61 (1H, d, H-3a, *J*=15.94)

**3´,5´-*O*-dicaffeoylquinic acid (2A):** UVmax, 326 nm, MS (ESI) m/z= 515.0, 353.1, (MS^2^: 191.2, 179.1), ^1^HNMR (d_3_-Acetonitrile), *δ* (ppm), 3.86 (1H, dd, H-4´), 5.35 (1H, m, H-5´), 5.41 (1H, m, H-3´a), 6.31 (1H, d, H-2a, *J*=15.90), 6.39 (1H, d, H-2b, *J*=15.97), 6.88 (2H, d, H-5a΄5b΄, *J*=8.14), 7.15 (2H, d, H-6a΄6b΄), 7.19 (2H, d, H-2a΄2b΄, *J*=1.97), 7.60 (1H, d, H-3b, *J*=15.93), 7.63 (1H, d, H-3a, *J*=15.84).

**4´,5´-*O*-dicaffeoylquinic acid** **(2B)**: UVmax, 326 nm, MS (ESI) m/z=515.0, 353.1, (MS^2^: 179.1, 173.1),  ^1^HNMR (d_3_-Acetonitrile), *δ* (ppm), 5.13 (1H, dd, H-4´), 5.61 (1H, m, H-5´), 4.38 (1H, m, H-3´a), 6.23 (1H, d, H-2a, *J*=16.00), overlapping (1H, d, H-2b), 6.82 (2H, d, H-5a΄5b΄), 6.99 (2H, d, H-6a΄6b΄), 7.15 (2H, d, H-2a΄2b΄), overlapping (1H, d, H-3b), 7.52 (1H, d, H-3a).
